# Immunosuppressive protocols with tacrolimus after cryopreserved aortal allotransplantation in rats

**DOI:** 10.1371/journal.pone.0201984

**Published:** 2018-08-09

**Authors:** Rudolf Spunda, Jan Hruby, Pavel Mericka, Mikulas Mlcek, Ondrej Pecha, Kathrin Splith, Moritz Schmelzle, Felix Krenzien, Jaroslav Lindner, Ivan Matia, Miroslav Spacek

**Affiliations:** 1 2nd Department of Surgery - Department of Cardiovascular Surgery, 1st Faculty of Medicine, Charles University in Prague and General University Hospital in Prague, Czech Republic; 2 Tissue Bank, Faculty Hospital Hradec Kralove, Charles University- Faculty of Medicine in Hradec Kralove, Hradec Kralove, Czech Republic; 3 Institute of Physiology, First Faculty of Medicine, Charles University in Prague; 4 Technology Centre of the Academy of Sciences of the Czech Republic, Prague, Czech Republic; 5 Department of Surgery, Campus Charité Mitte and Campus Virchow-Klinikum, Charité-Universitätsmedizin Berlin, Berlin, Germany; 6 Department of Cardio-Vascular Surgery, Hospital Hietzing and Karl Landsteiner Institute for Cardio-Vascular Research, Vienna, Austria; University of Toledo, UNITED STATES

## Abstract

**Objectives and design:**

The aim of our study was to simulate in rats all aspects and techniques used in our new clinical program of cryopreserved alloarterial transplantation and investigate the influence of two immunosuppressive protocols with tacrolimus on acute rejection of these allografts.

**Materials and methods:**

Cryopreserved abdominal aortic grafts were transplanted between Brown-Norway and Lewis rats. Tacrolimus (0.2 mg/kg daily) was administered from day 1 to day 30 (TAC1) or from day 7 to day 30 (TAC7), respectively. No immunosuppressed isogeneic (ISO) and allogeneic (ALO) rats combination served as control. Aortal wall infiltration by immunocompetent cells (MHC II+ cells of recipient origin) was studied on day 30 after transplantation. Flow cytometry was used for the analysis of day 30 sera for the presence of donor specific anti-MHC class I and II antibodies.

**Results:**

The aortal allografts in both immunosuppressed groups showed regular morphology of aortal wall with no depositions of immunoglobulin G on day 30. The adventitial infiltration of non-immunosuppressed aortal allografts by MHC class II positive cells of recipient origin was significantly higher (ALO 20.7±6.7 cells, P<0.001) compared to both immunosuppressed groups (TAC1 5.9±5.5 cells, TAC7 6.1±5.1 cells). Day 30 sera from the allogeneic non-immunosuppressed animals decreased significantly the binding of fluorescence-labelled MHC class I (46.9±19.4%) and class II (65.8±11.9%) antibody to donors spleen cells compared with day 30 sera from both immunosuppressed groups (TAC1, anti-MHC class I 102.4±4.2%, *p* < 0.001, anti-MHC class II 102.6±6.0%), (TAC7, anti-MHC class I 79.9±3.3%, *p* < 0.001, anti-MHC class II 80.9±2.7%).

**Conclusion:**

Both immunosuppressed protocols with tacrolimus (administration from day 1 or from day 7 following transplantation) were able to suppress acute cell- and antibody-mediated rejection of cryopreserved abdominal aortic allografts processed in accordance with our new standardized clinical protocol.

## Introduction

One of the most dangerous complication in vascular surgery is infection of vascular protheses or stentgrafts [1]. Contemporary therapy of choice in this life-threatening condition is reoperation with replacement of an infected foreign material with an arterial allograft [2]. The use of cold-stored arterial grafts (conserved in storage medium by 4 °C) in the treatment of this complication was successfully introduced by Kieffer in Paris in the late eighties of 20^th^ century [3]. Few years later was these method introduced in the Czech Republic as well [4,5].

However the implication of the Directive 2004/23/EC of the European Parliament and of The Council on Setting Standards of Quality and Safety for the Donation, Procurement, Testing, Processing, Preservation, Storage and Distribution of Human Tissues and Cells led to the cessation of the further use of cold-stored arterial allografts in this indication in many European countries including France [1] and Germany [6] In accordance with this trend we introduced the clinical program of cryopreserved alloarteries transplantation in the Czech Republic in 2011 [7].

After transplantation of cold-stored and cryopreserved arteries we can observe immune reaction similar to the rejection process in solid organ transplantation [8,9]. In patients after transplantation of arterial allografts with no immunosuppression we can find a higher incidence of graft related complication as graft ruptures, graft aneurysm formation or thrombosis [2]. On the other hand there is a good long term patency rates and no aneurysmal formation in patients after simultaneous organ and arterial transplantation with triple immunosuppression postoperatively [10,11,12].

Even though immunosuppression can improve results after allograft transplantation this therapy is not generally accepted. This is probably caused by wariness to decrease immune reaction in patients with florid infection [9,13].

In our previous experimental work we considered a positive effect of low-dose tacrolimus immunosuppression on the inhibition of acute cell- and antibody-mediated rejection of cold-stored arterial grafts in rats [13,14]. In addition, we confirmed a possibility of delaying the administration of low-dose tacrolimus after an arterial transplantation for 1 week without any negative influence on both types of rejection [14]. These conclusions led to introduction of new standardized immunosuppressive protocols with low-dose of tacrolimus in patients after cold-stored arterial transplantations in the Czech Republic [4].

Therefore, the aim of our study was to simulate in rats all aspects and techniques used in our clinical program of cryopreserved alloarterial transplantation and investigate influence of two immunosuppressive protocols with tacrolimus on the cell-mediated and antibody-mediated rejection of cryopreserved arterial allografts processed by this protocol.

## Material and methods

### Ethics statement

This study was carried out in strict accordance with the recommendations in the Guide for the Care and Use of Laboratory Animals of the National Institutes of Health. The protocol was approved by the Committee on the Ethics of Animal Experiments of Charles University, 1^st^ Medical Faculty (no. UK 1.LF 563/13, MSMT-14808/2014-6)

### Animals

Adult male inbred Brown-Norway (BN; RT1^n^) and Lewis (LEW; RT1^l^) rats were obtained from Charles River (Sulzfeld, Germany). Principles of laboratory animal care were followed and all rats were maintained according to the National Institute of Health Guidelines. Male LEW rats (n = 24, 191–250 g) were used as recipients of allogeneic or syngeneic cryopreserved abdominal aortal grafts. Male BN rats (n = 9, 191–254 g) were used as donors of allogeneic cryopreserved abdominal aortal grafts. Male LEW rats (n = 3, 248-254g) were used as donors of syngeneic cryopreserved abdominal aortal grafts. Each transplanted animal was held in a separate cage during the 30-day follow-up period.

Only animals that passed the whole follow-up period were included into the study.

### Operative procedure of donor animals

The donor animals were anaesthetized by an intramuscular injection of ketamine (Narkamon^®^, Spofa a.s., Prague, Czech Republic) at 100 mg/kg and xylazine (Rometar^®^, Spofa a.s., Prague, Czech Republic) at 10 mg/kg. A 1,5–2,0 cm long segment of the infrarenal aorta was excised after the administration of heparin (100 IU/kg) via the inferior caval vein. The graft was gently flushed with 2 ml of Celsior^®^ solution (Genzyme, Netherlands) containing 100 IU/ml of heparin, and prepared for cryoconservation as described below. The animals were then euthanized by intracaval administration of a lethal dose of thiopental (Thiopental^®^, Spofa a.s., Prague, Czech Republic). There were excluded 2 recipients animals for not passing the whole follow up period. Both of them died on 1. postoperative day. The necropsy showed no surgical cause of death.

### Protocol of cryoconservation

Cryoconservation of aortal grafts was performed by methods used in clinical practice of the Tissue Bank of The University Hospital Hradec Kralove in the Czech Republic. This Tissue Bank is fully licensed by the Czech national competent authority [15,16] and fully integrated to the clinical program of cryopreserved alloarteries transplantation in the Czech Republic since 2011 [7]. The clinical methods were modified to correspond with the conditions of work in an experimental operating room in which all bellow described procedures were performed. Only high quality material and drugs for human use meeting the requirements of the Directive of the European Parliament and Council No. 23/2004/EC were used by these procedures.

The 6–8 donor animals were operated at once. The flushed aortal graft of each donor animal was immediately after excision placed into pre-cooled Celsior^®^ conservation solution (Genzyme, The Netherlands). The grafts were then stored at the temperature of melting ice in closed sterile certified plastic jars (Medfor 250 ml Farnborough, UK) until finalization of all donor´s operations. The aortal grafts were subsequently put into double sterile disposable plastic bags (CryoMACS Freezing Bag 500, Miltenyi Biotec GmbH, Germany) containing 25ml of pre-cooled 6% solution of hydroxyethylstarch m.w. 130.000 Da (Voluven 6%, Fresenius Kabi, Germany). Each bag contained two aortal grafts from two different donors. The content of the bag was subsequently mixed with the pre-cooled 20% dimethylsulfoxide (WAK ChemieMedical GmbH, Germany) in the 6% hydroxyethylstarch solution (Voluven 6%, Fresenius Kabi, Germany). The plastic bags were closed by sealing at the sealing machine (STERISEAL B 83-R, Cevor s.r.o., Troubsko u Brna, Czech Republic), put into outer metal cassettes (ST 100, Consarctic GmbH, Schölkrippen, Germany) and stored at the temperature of melting ice until freezing. The controlled–rate freezing by the rate of -1K/min to -90°C, -5K/min to -150°C was performed at the programmable freezer (Kryo-10, Planer Biomed, Sunbury on Thames, England). + The aortal grafts were stored in liquid nitrogen vapour phase by temperature of -190°C until implantation.

### Thawing protocol

The outer metal cassettes with bags containing the aortal grafts were transported at the day of implantation in liquid nitrogen vapour phase from the Tissue Establishment of the Institute of Haematology in Prague to the operating room of Institute of Physiology of 1^st^ Medical faculty, Charles University in Prague. In the operating room were the cassettes removed from the shipper and placed for about 60 minutes into a refrigerator with temperature of +4°C and then were kept for another 60 minutes by the room temperature. Subsequently, the aortal grafts were removed from the bag and each of two grafts was divided into two pieces of an identical length to be used in two rat recipients. After dividing was each aortal graft stored separately in 10 ml of Custodiol solution (Custodiol^®^, Dr. Franz Köhler Chemie GmbH, Germany) in the refrigerator with temperature of +4°C until the beginning of anastomosis in the recipient animal.

### Operative procedure of recipient animals

The recipient animals were anaesthetized with intramuscular injection of sufentanil (Sufenta^®^, Janssen Pharmaceutica Inc., Beerse, Belgium) at 20 μg/kg and azaperone (Stresnil^®^, Janssen Pharmaceutica Inc., Beerse, Belgium) at 1 mg/kg to ensure more natural awakening.

Following a midline laparotomy, the aortal grafts were transplanted orthotopically into the recipient’s infrarenal aorta with an interrupted 10/0 mono-filament suture (Ethicon Inc., Sommerville, New Jersey, USA). Neither anti-coagulants nor anti-platelet drugs were administrated during the transplantation or in the postoperative period.

### Animal groups

The recipient animals of cryopreserved aortal grafts were divided into four groups according to the postoperative immunosuppressive protocol: group ISO was that of isogeneic control (LEW to LEW, n = 6, 191–250 g), group ALO was that of allogeneic control (BN to LEW, n = 6, 193–245 g) with no administration of tacrolimus; animals in group TAC1 (BN to LEW, n = 6, 228–242 g) were immunosuppressed from day 1 to day 30; and animals in group TAC7 (BN to LEW, n = 6, 195–212 g) were immunosuppressed from day 7 to day 30 after the transplantation.

### Immunosuppressive therapy

Two different protocols of tacrolimus immunosuppression (day 1–30 in the group TAC1 and day 7–30 in the group TAC7, respectively) were used in animals after allogeneic cryopreserved aortal transplantation. Tacrolimus (Prograf^®^; Astellas Pharma Inc., Tokyo, Japan) suspended in saline solution was administered intramuscularly in daily doses of 0.2 mg/kg. Pure saline solution in equivalent volume was administered intramuscularly daily for first 6 days in group TAC7 animals and for 30 days in the non-immunosuppressed animals in group ISO and ALO.

### Blood samples

Blood samples for determination of donor specific anti MHC antibodies on day 0, and day 30 in all groups and for determination of tacrolimus concentration on day 30 in immunosuppressed groups TAC1 and TAC7 were collected by orbital sinus puncture as described by van Herck [17]. The animals were anaesthetized by an intramuscular injection of ketamine (Narkamon^®^, Spofa a.s., Prague, Czech Republic) at 100 mg/kg and xylazine (Rometar^®^, Spofa a.s., Prague, Czech Republic) at 10 mg/kg during this procedure.

### Arterial grafts explantation on day 30

The animals were anaesthetized by an intramuscular injection of ketamine (Narkamon^®^, Spofa a.s., Prague, Czech Republic) at 100 mg/kg and xylazine (Rometar^®^, Spofa a.s., Prague, Czech Republic) at 10 mg/kg on day 30. After the blood samples collection (see above) a midline re-laparotomy was performed. Aortal grafts were excised after the administration of heparin (100 IU/kg) via the inferior caval vein in all experimental groups. The animals were then euthanized by intracaval administration of a lethal dose of thiopental (Thiopental^®^, Spofa a.s., Prague, Czech Republic).

### Parameters under study

#### Animal weight

The animals body weight in all experimental groups was determined daily before intramuscular injection of saline solution with or without tacrolimus.

### Concentration of tacrolimus in peripheral blood

In animals of group TAC1 and TAC7, blood levels of tacrolimus were evaluated with an enzyme enhanced immunoassay technique (Emit^®^ 2000 Tacrolimus assay, Dade Behring Inc., Deerfield, Illinois, USA) on day 30 after transplantation.

### Histologic analysis of explanted arterial grafts

The explanted aortal grafts were embedded in Sakura Finetek Tissue Tek Cryomold holders (Sakura Finetek, Tokyo, Japan) and Sakura Finetek Tissue Tek O.C.T. compound (Sakura Finetek, Tokyo, Japan). The samples were frozen in 2-methylbutane (Fluka Chemika, Buchs, Switzerland), cooled with liquid nitrogen, and stored until processed at −80°C.

The sections for histological analysis were taken from the midportion of the graft to avoid tissue that may have reacted to the suture material. The 5 μm thick cross sections were stained with a Hematoxylin & Eosin and a Van Gieson with elastica stain.

### Immunohistochemical analysis of explanted arterial grafts

The sections were taken from the midportion of the graft to avoid tissue that may have reacted to the suture material.

#### Detection of CD4+ cells, CD8+ cells and Von Willebrand factor

Immunohistochemistry was performed on 8 μm thick sections with a two-step indirect method. Briefly, the sections were fixed in cold acetone for 10 minutes. After rinsing in 0.2% Triton X 100 and phosphate-buffered saline, the specimens were incubated with a primary antibody (anti-CD4 (W3/25, Cymbus Biotechnology LTD, Hampshire, UK), anti-CD8 (OX-8, Cymbus Biotechnology LTD, Hampshire, UK), anti-Von Willebrand factor (Dako Denmark A/S, Glostrup, Denmark) for 60 min. Endogenous peroxidase was blocked by incubating in 0.3% H_2_O_2_ and 70% methanol for 30 minutes. Next, the sections were incubated with a secondary antibody (Histofine^®^ Simple Stain Rat MAX PO, Nichirei, Japan) for 30 min, then incubated with Dako Liquid DAB+ Substrate-Chromogen System (Dako Denmark A/S, Glostrup, Denmark) for 5 min. The specimens were counterstained and dipped in Entellan (Merck KGaA, Darmstadt, Germany).

The slides were then scored in a blinded fashion. Cells were counted at 5 locations at x1000 magnification. The cellularity was defined as the mean value of the cells counted.

#### Detection of Lewis MHC class II positive cells

Immunohistochemistry was performed on 8 μm thick sections with a three-step indirect method. Briefly, the sections were fixed for 10 minutes in cold acetone. After sections were rinsed in 0.2% Triton X 100 and phosphate-buffered saline, endogenous biotin was blocked with the Biotin blocking system (Dako Denmark A/S, Glostrup, Denmark). The tissues were then incubated in 10% horse serum to prevent unspecific binding, and then a primary antibody was applied for 60 min. Then, endogenous peroxidase was blocked in 0.3% H_2_O_2_ and 70% methanol for 30 minutes. The specimen was incubated with a secondary biotinylated horse anti-mouse antibody (Vector Lab, Burlingame, California, USA), followed by an incubation with R.T.U. Vectastain Elite ABC Reagent (Vector Lab, Burlingame, California, USA). Finally, specimens were incubated for 5 min with Dako Liquid DAB+ Substrate-Chromogen System (Dako Denmark A/S, Glostrup, Denmark), counterstained, and dipped in Entellan (Merck KGaA, Darmstadt, Germany).

The slides were then scored in a blinded fashion. Cells were counted at 5 locations at x1000 magnification. The cellularity was defined as the mean value of the cells counted.

### Detection of immunoglobulins

After processing, the 8-μm thick sections were rinsed in PBS and air-dried. The tissues were then incubated with primary antibody directly conjugated with fluorescein isothiocyanate (Chemicon International Inc, Temecula, California, USA) for 30 min. The specimens were then dipped in glycerine medium and immediately analysed under a fluorescence microscope.

### Flow cytometry analysis of blood samples

Brown-Norway splenocytes were thawn, washed in phosphate-buffered saline (PBS) and resuspended in PBS with 1% fetal bovine serum (FBS). 100.000 cells were incubated for 30 min at 4°C with 10 μL of recipient serum. Cells were washed twice in PBS (1% FBS) and then incubated with original antibodies as follows: MHC expression on quiescent BN splenocytes was evaluated using a Biotin-MHC class I (anti-RT1.Ac, OX-27, Acris Antibodies GmbH, Herford, Germany) or a Biotin-MHC class II (anti-RT1.D, OX-17, BD Biosciences, Heidelberg, Germany) primary antibody and a PE-Cy7-Streptavidin secondary antibody (BD Biosciences, Heidelberg, Germany). 10.000 cells were acquired on a FACSCanto II flow cytometer (BD Biosciences, Heidelberg, Germany) and analyzed using FACSDiva^™^ software (BD Biosciences, Heidelberg, Germany). Graphic presentation in histograms allowed the determination of mean fluorescence intensity on a log scale. MHC class I or class II antibody binding on the cells without previous recipient´s serum incubation was set to 100%. The serum antibodies from allografted LEW rats, when presented, were bound comparatively to MHC class I and MHC class II molecules on BN splenocytes. The inhibition of the fluorescence-labelled MHC class I and II antibody binding consequently decreased the measured fluorescence signal.

### Statistical analysis

The values in the text and tables are expressed as the mean±standard deviation (SD). Bar charts in graphs represent means and whiskers symbolize 95% confidence intervals (CI) in all graphs. Comparisons of parameters under study between experimental groups (weight increase, CD4+ cells, CD8+ cells, MHC II+ cells, anti MHC I and anti MHC II antibodies) were performed using the analysis of variance (ANOVA), followed by the Tukey HSD Multiple Comparisons test. All analyses were conducted in Stata (version 12.1). All charts were accomplished using the Data-Driven Documents library in JavaScript (D3.js, version 3.0).

## Results

### Animals

**Fig 1:**
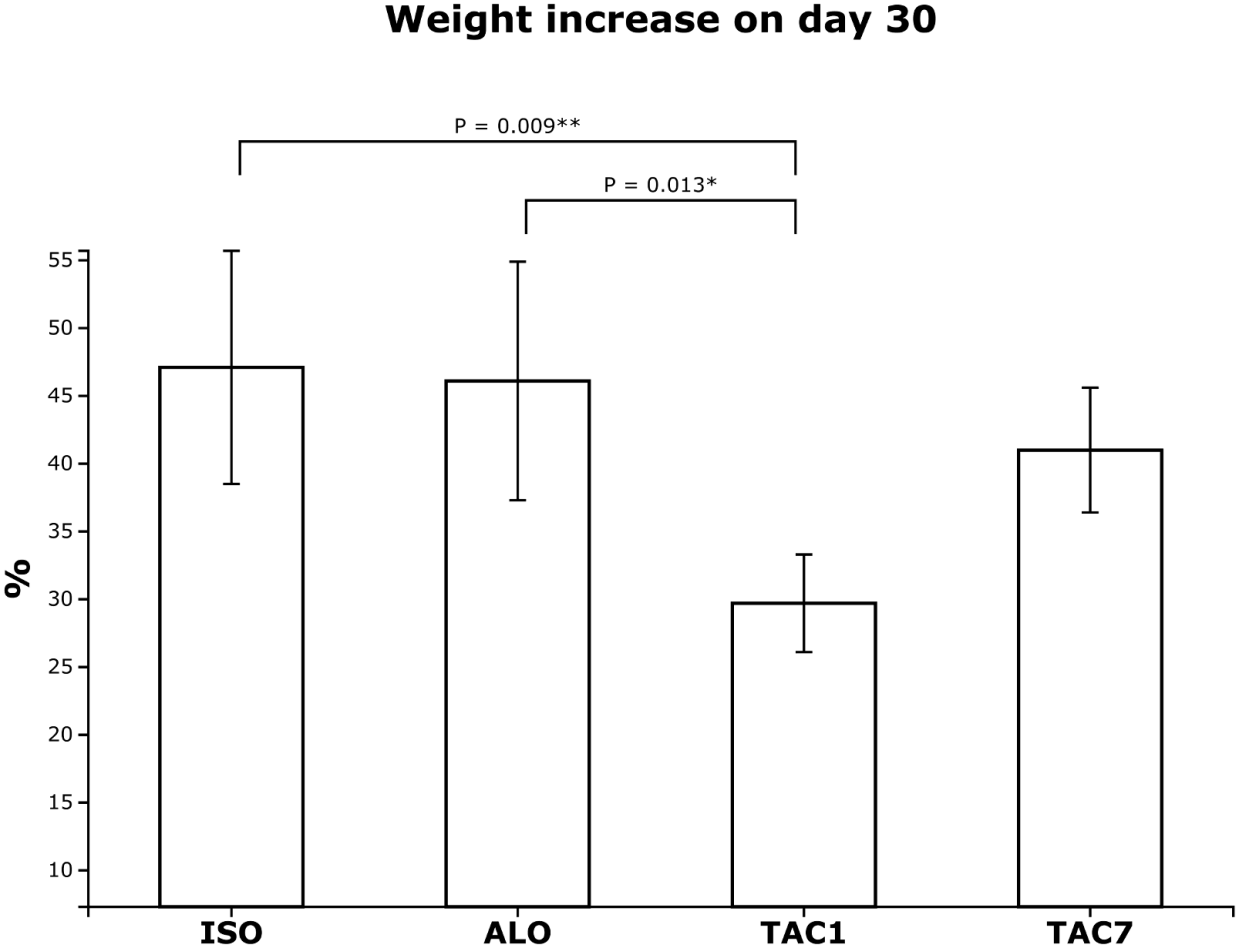
Weight increase on day 30 after transplantation of aortic grafts expressed as a percentage of preoperative weight. No immunosuppressed animals (group ISO, group ALO) showed significantly higher weight increase on day 30 when compared to from day 1 immunosuppressed animals (group TAC1). However, the weight increase in from day 7 immunosuppressed animals (group TAC7) was compared to animals without immunosuppression (group ISO, group ALO).

An increase from pre-operative weight was observed in all groups on day 30. The weight increase expressed as a percentage of preoperative weight was significantly higher (P< .05) in both no immunosuppressed groups (group ISO +47.1±10.5%, group ALO +46.1±10.8%) compared to from day 1 immunosuppressed animals in group TAC1 (+29.7±4.4%). The weight increase (+41.0±5.6%) in from day 7 immunosuppressed animals (group TAC7) was compared to both no immunosuppressed groups (Fig 1) (S1 Table).

### Immunosuppression with tacrolimus

Measurement for blood concentrations of tacrolimus were performed in order to assure adequate tacrolimus concentrations of 3–7 ng/mL by daily doses of 0.2 mg/kg. The mean tacrolimus blood level on day 30 in from day 1 immunosuppressed animals of group TAC1 was slightly higher (4.6±0.7 ng/mL) compared to from day 7 immunosuppressed animals of group TAC7 (4.0±0.2 ng/mL) (P = 0.035) (Table 1). No adverse effects of the tacrolimus administration, such as diarrhea or a persisted weight loss, were observed in any of the experimental animals.

**Table 1:** Basic characteristics of experimental groups.

Group	Characteristic	Immunosuppression	Cryoconservation (days)	Weight increase on day 30 (%)	Tacrolimus blood level on day 30 (ng/mL)
ISO	LEW to LEW	no	172.7±2.6	47.1±10.5	no
ALO	BN to LEW	no	179.3±2.6	46.1±10.8	no
TAC1	BN to LEW	Tacrolimus day 1–30	180.2±6.9	29.6±4.4	4.6±0.7
TAC7	BN to LEW	Tacrolimus day 7–30	180.3±0.5	40.1±5.6	4.0±0.2

LEW—male Lewis rats

BN—male Brown-Norway rats

### Histology and immunohistology

**Fig 2:**
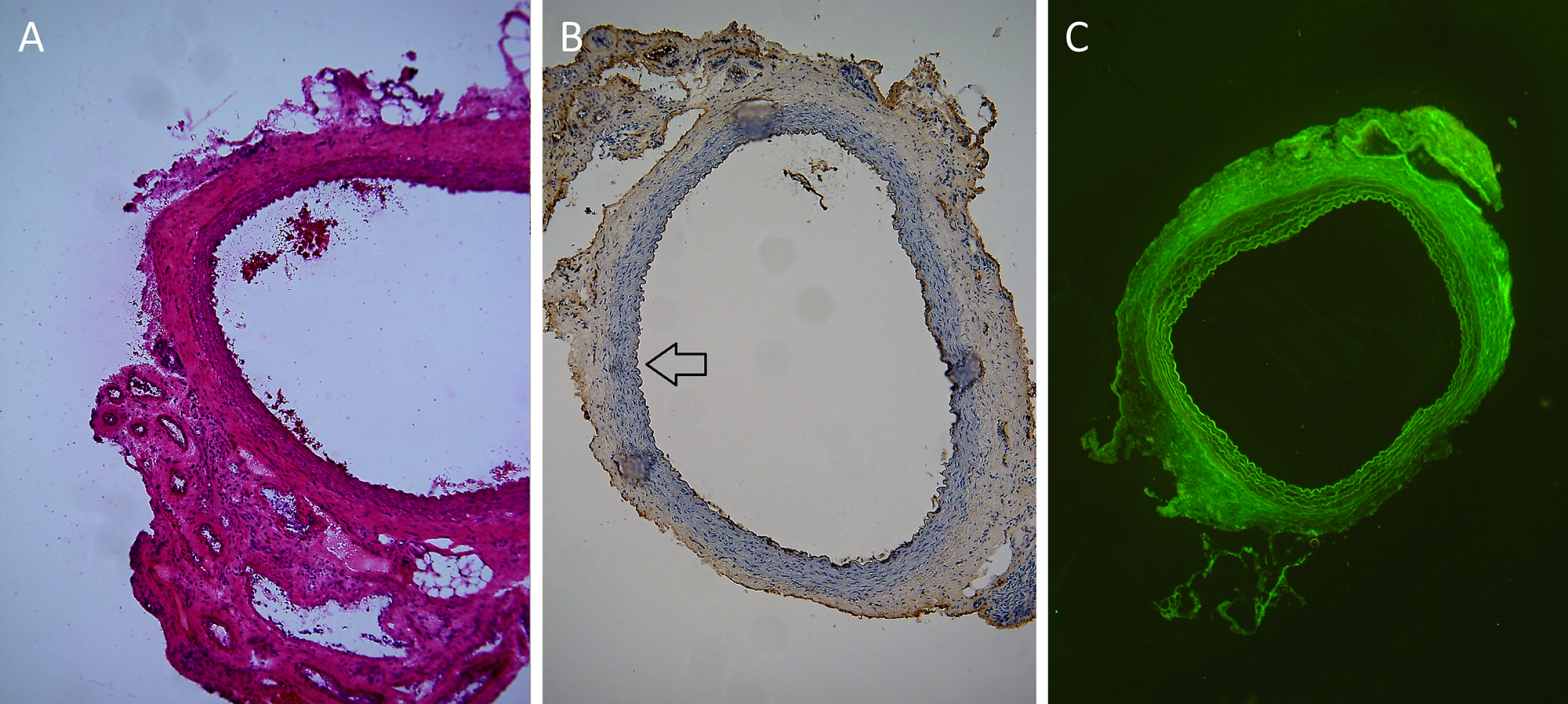
a, b, c. Representative light microscopic features of immunosuppressed cryopreserved aortal allografts obtained at 30 days following transplantation. a—The allografts showed normal histological feature of abdominal aorta with clear differentiation of all three basic anatomical layers, with no signs of intimal hyperplasia, smooth muscle cells necrosis or higher adventitial cellular infiltration. (Haematoxilin-Eosin, original magnification x 100). b—The luminal surface of allografts (arrow) was covered by monolayer of endothelial cells (stained brown). (Anti-Von Willebrand factor antibody, original magnification x 100). c—No deposition of immunoglobulins G was detected in the medial or intimal layer of cryopreserved allografts. (Anti-IgG fluorescein isothiocyanate-conjugated antibody, original magnification x 40).

The aortal grafts in both tacrolimus immunosuppressed groups (TAC1, TAC7) on day 30 showed regular morphology of aortal wall with clear differentiation of all three basic anatomical layers—tunica intima, media and adventitia. The luminal surface of intima was covered by a monolayer of endothelial cells. There were no signs of necrosis or destruction of tunica media. No IgG depositions were detected in tunica media in both immunosuppressed groups. The infiltration of tunica adventitia with mononuclear cells was comparable to isogeneic grafts of group ISO (Fig 2a, 2b and 2c). The allografts showed normal histological feature of abdominal aorta with clear differentiation of all three basic anatomical layers, with no signs of intimal hyperplasia, smooth muscle cells necrosis or higher adventitial cellular infiltration.

**Fig 3:**
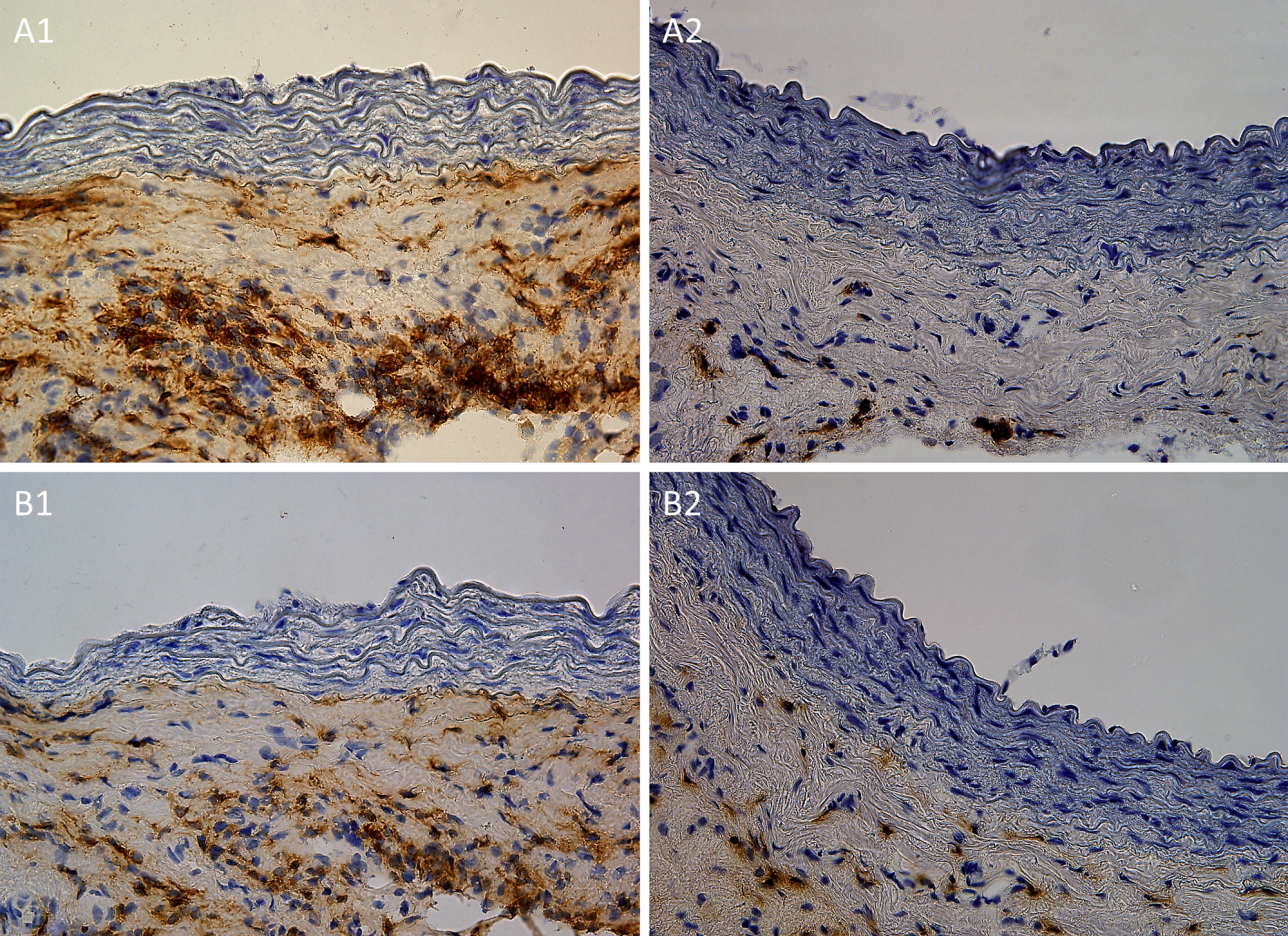
a1, a2, b1, b2. Representative light microscopic histological features of adventitial infiltration of cryopreserved allografts by mononuclear cell at 30 days following transplantation. The adventitial infiltration of Brown-Norway cryopreserved aortal grafts by MHC class II positive cell of Lewis origin (stained brown) (Fig 3 a1) was significantly reduced by both types of immunosuppressive protocols with tacrolimus (Fig 3 a2). The adventitial infiltration of Brown-Norway aortal grafts by CD4+ cell (Fig 3 b1) was significantly reduced by both immunosuppressive protocols with tacrolimus as well (Fig 3 b2). Original magnification ×400.

Surprisingly the histology of allogeneic no immunosuppressed cryopreserved aortal grafts of group ALO showed regular morphology of aortal wall as well. All three basic layers were well preserved as well. No IgG depositions were found in muscular layer of allogeneic grafts. However, the infiltration of tunica adventitia with mononuclear cells was higher compared to isogeneic and both immunosuppressed groups. (Fig 3)

### Lewis MHC class II + cells, CD4+ cells, CD8+ cells in tunica adventitia

The adventitial infiltration by CD4+, CD8+, and Lewis MHC class II positive cells in both immunosuppressed groups (group TAC1 and TAC7) on day 30 was comparable to the adventitial infiltration of isogeneic grafts in the group ISO.

The absence of immunosuppression in group ALO led to significantly higher infiltration of tunica adventitia with immunocompetent cells compared to all other groups (Table 2, Fig 3).

**Table 2:** Assessments of rat cryopreserved aortic graft on day 30 after transplantation into the abdominal aorta.

Group	Characteristic	IS	Intima		Media		Adventitia*		
			Endothelial layer	Intimal hyperplasia	IgG deposition	SMC necrosis	LEW MHC class II+ cells**	CD8+ cells	CD4+ cells
ISO	LEW to LEW	no	+	-	-	-	6.3±4.4	2.2±2.7	3.9±2.6
ALO	BN to LEW	no	+	-	-	-	20.7±6.7^1^	6.9±5.4^1^	9.6±6.5^1^
TAC1	BN to LEW	Tac 1–30	+	-	-	-	5.9±5.5	3.5±3.3	2.3±1.6
TAC7	BN to LEW	Tac 7–30	+	-	-	-	6.1±5.1	3.1±3.8	2.0±1.5

^1^ The total amounts of Lewis MHC class II positive, CD8-positive and CD4-positive cells in tunica adventitia in group ALO were significantly higher (P<0.001) than those observed in all the other groups (ISO, TAC1, TAC7).

+ positive finding, − negative finding

* Numbers of Lewis anti MHC class II, CD8-positive and CD4-positive cells in one microscopic field viewed at a magnification of x1000

** Major histocompatibility complex class II positive cells of Lewis origin

IS—immunosuppression

Tac—tacrolimus

### Anti MHC class I antibodies in serum

**Fig 4:**
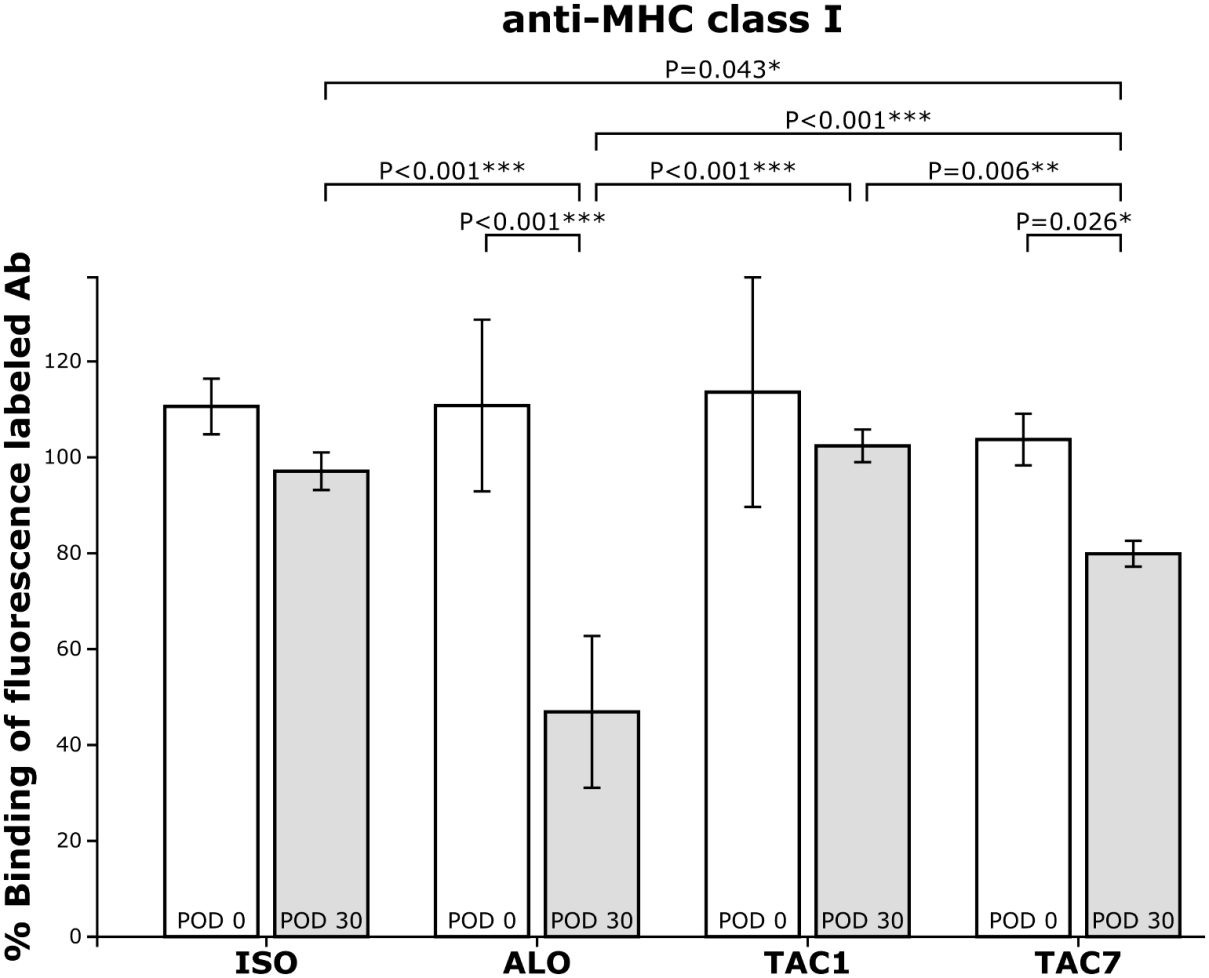
Anti MHC class I antibodies in serum. The percentage of binding of the anti-MHC class I antibody (anti-RT1.Ac, OX-27) to quiescent BN splenocytes in the presence of sera obtained from recipient rats pretransplant (POD 0) and on day 30 after transplantation (POD 30). ISO—isogeneic group with no immunosuppression. ALO—allogeneic group with no immunosuppression. TAC1—from day 1 immunosuppressed allogeneic group. TAC7—from day 7 immunosuppressed allogeneic group.

Sera of isogeneic animals (group ISO, 97.1±4.7%) and from day 1 immunosuppressed animals (group TAC1, 102.4±4.2%) obtained on day 30 showed no change of inhibition of the binding of fluorescence-labeled MHC class I antibody to BN-splenocyte compared to pretransplant sera (group ISO, 110.6±7.1%, group TAC1, 113.6±29.3%) (Fig 4).

Sera from day 7 immunosuppressed animals (group TAC7) obtained on day 30, showed higher inhibition of the fluorescence-labeled MHC class I antibody binding to BN-splenocyte (79.9±3.3%) compared to pretransplant sera (103.7±6.6%, p = 0.026) (Fig 4).

Sera of allogeneic non-immunosuppressed animals (group ALO) obtained on day 30 after transplantation significantly decreased the binding of fluorescence-labeled MHC class I antibody to BN spleen cells (46.9±19.4%), compared to pretransplant sera (110.8±21.9%, *p* < 0.001) (Fig 4).

In addition, sera from the allogeneic non-immunosuppressed animals (group ALO) obtained on day 30 showed significant inhibition of fluorescence-labelled MHC class I antibody binding to BN spleen cells (46.9±19.4%), compared with day 30 sera from the isogeneic group (group ISO, 97.1±4.7%, *p* < 0.001), as well as with both immunosuppressed groups (group TAC1, 102.4±4.2%, *p* < 0.001), (group TAC7, 79.9±3.3%, *p* < 0.001) (Fig 4).

### Anti MHC class II antibodies in serum

**Fig 5:**
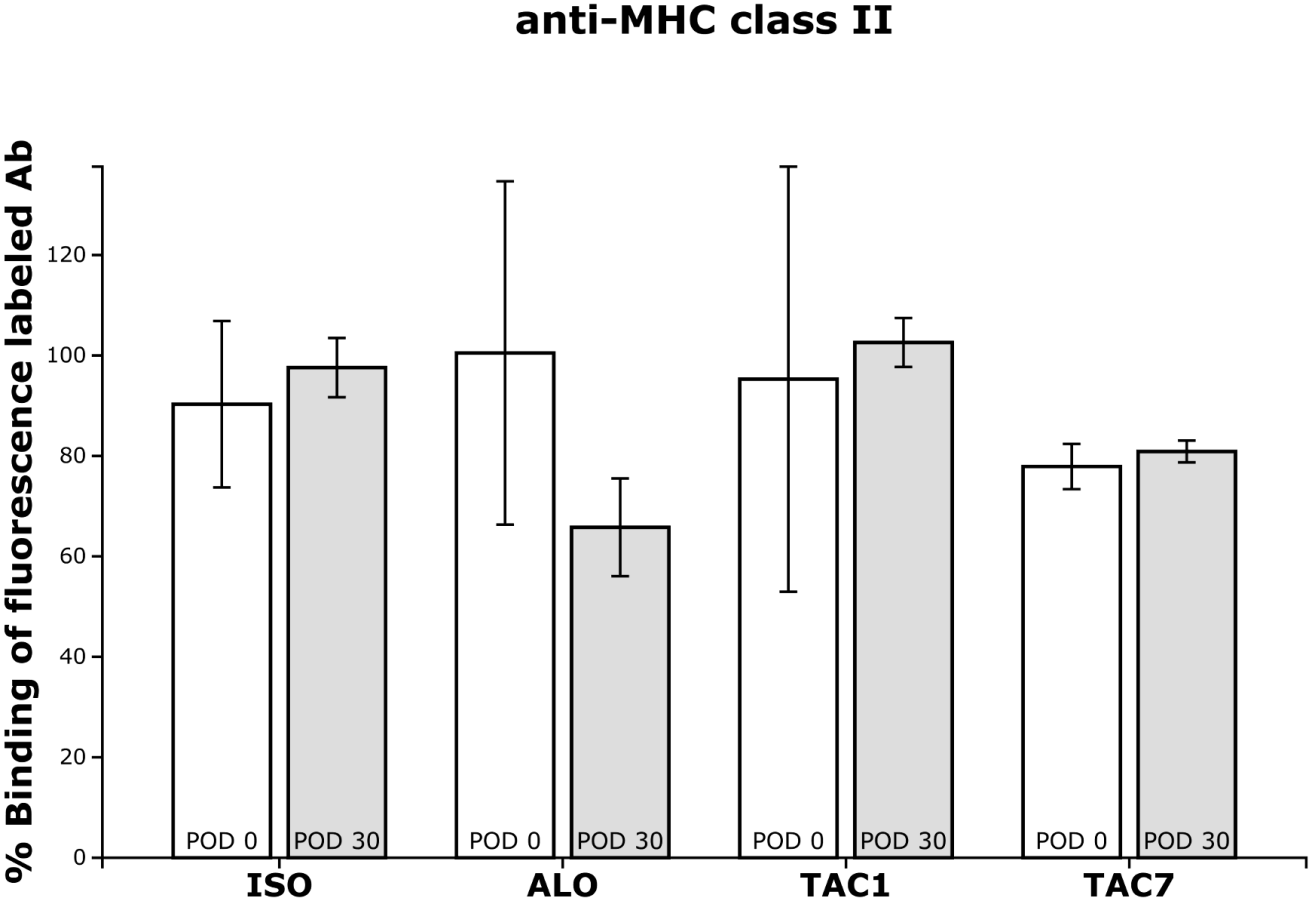
Anti MHC class II antibodies in serum. The percentage of binding of the anti-MHC class II antibody (anti-RT1.D, OX-17) to quiescent BN splenocytes in the presence of sera obtained from recipient rats pretransplant (POD 0) and on day 30 after transplantation (POD 30). ISO—isogeneic group with no immunosuppression. ALO—allogeneic group with no immunosuppression. TAC1—from day 1 immunosuppressed allogeneic group. TAC7—from day 7 immunosuppressed allogeneic group.

Isogeneic group sera (group ISO) as well as both allogeneic immunosuppressed groups sera (group TAC1 and TAC7) showed no inhibition of the fluorescence-labelled MHC class II antibody binding to BN-splenocyte during the entire follow-up period (Fig 5). By contrast, only sera from allogeneic non-immunosuppressed animals (group ALO) obtained on day 30 after transplantation showed inhibition (65.8±11.9%) of fluorescence-labelled MHC class II antibody binding to BN-splenocyte. However, this inhibition was not statistically significant compared to pretransplant values in this group (100.5±41.9%) (Fig 5).

## Discussion

The present study examined the effect of two immunosuppressive protocols with low dose tacrolimus on rejection of cryopreserved abdominal aortic allografts in rats. Rat allografts were processed in accordance to standardized cryoconservation and implantation protocol routinely used in the clinical setting in the Czech Republic since 2011. Both immunosuppressed protocols with tacrolimus (administration from day 1 or from day 7 following transplantation) were able to suppress cell-mediated and antibody-mediated rejection of cryopreserved abdominal aortic allografts during the 30 day follow-up period.

The model of rat or mice aortal allotransplantation is routinely used in experimental transplant medicine [18] and is closely related to the development of new immunosuppressive drugs since the 1960s. The immunosuppressive protocols with azathioprine [19], azathioprine and prednisone [20], 6-mercaptopurine [21], cyclosporine A [22], sirolimus [23], cyclosporine with methylprednisolone and azathioprine [24], mycophenolat mofetil [25], tacrolimus [18], 15-deoxyspergualin [26], cyclosporine A and FTY720 [27], tacrolimus and FR260330 [28], everolimus [29], everolimus with clopidogrel [30] were confirmed to be able to suppress to some degree the immune-mediated destruction of arterial allografts.

Despite of it, is the use of immunosuppression in patients after clinical arterial allograft implantation not generally accepted by vascular surgeons [1]. This is probably caused by no acceptance of the antigenicity of arterial allografts by vascular surgeons [9] and/or a reluctance to use immunosuppressive treatment in patients with ongoing infection [31]. However, if the immunosuppressive therapy after clinical arterial transplantation is used, the drug most commonly used is cyclosporine A [32].

Recently, the most complex view on the use of cyclosporine A in patients after in situ revascularisation with cold-stored arterial allograft in the treatment of aortic graft infection represents the prospective, comparative, single-centre study published in 2011 by Pupka et al. [2]. One group of patients was immunosuppressed with cyclosporine A. Second group of patients had because of their own decision no immunosuppression. The immunosuppressive protocol was based on the administration of cyclosporine A on daily doses of 1–3 mg per kg of body mass with serum concentration of 140–150 mg/L. The drug was taken until the end of the study (mean follow up 22.8 months). The graft thrombosis (12%), rupture of the graft (12%) with death of the patient (8%) and graft aneurysm (8%) was observed only in non-immunosuppressed group. No adverse effects of immunosuppression were reported in this study [32].

However, published data confirm considerable vascular [33] and metabolic [34] side effects with the use of cyclosporine A. Indeed, in rat cardiac allografts led cyclosporine A to the increase of transplant arteriosclerosis by the up-regulation of the expression of tissue growth factor beta (TGF-b) [35]. In rat aortic allografts led the therapy with cyclosporine A to endothelialitis and accelerated arteriosclerosis [36]. In addition, the mice murine aortic allografts study published by Eckl et al. also revealed that monotherapy with cyclosporine is not sufficient in preventing the formation of transplant arteriosclerosis [29].

An attractive alternative to cyclosporine A represents tacrolimus. Tacrolimus, a macrolide compound isolated from Streptomyces tsukubaensis, is 10 to 100 times more potent than cyclosporine A [37]. In clinical kidney transplantation resulted tacrolimus immunosuppression when compared with cyclosporine therapy in significantly reduced risk of graft failure, without an increase in the incidence of adverse events associated with long-term immunosuppression [38]. Moreover, it was proven that tacrolimus (5.0 mg/kg/day, intramuscular application, blood concentrations not determined) was able to enhance the viability and cellular integrity characteristics of the donor cells in cryopreserved thoracic aortas transplanted between Brown-Norway and Lewis rats [39].

All these facts about immunogenicity of arterial allografts, tacrolimus-based immunosuppression and our good results with triple immunosuppression in patients after simultaneous organ and vascular transplantation [40] led our group to study the immunosuppressive protocol with tacrolimus after rat cold-stored abdominal aortic transplantation in 2002 [13]. Subsequently in 2004, we started to use in patients after replacement of infected vascular prosthesis or stentgraft with cold-stored arterial allograft a standardized immunosuppressive protocol consisting of orally administered tacrolimus [4]. The drug is administered from day 7 after transplantation and is given throughout the entire period of allograft patency. The usual starting dose is 6 mg/day. The usual maintenance daily dose is 2 mg/day, respectively. The tacrolimus blood levels are determined periodically with blood concentration range between 4 and 7 ng/mL. No significant adverse effects of immunosuppression were observed in these patients.

In the present experiment we didn´t observed any clinically significant adverse effects of immunosuppression with tacrolimus given intramusculary in daily doses of 0.2 mg/kg/day. However, the postoperative weight increase in rats with administration of tacrolimus from day 1 after transplantation was significantly lower as in rats without immunosuppression or with delayed administration of immunosuppression.

In the study of tacrolimus induced hypertension of Takeda et al [41] the rats have obtained tacrolimus (5.0 mg/kg/day, peroral application, blood concentrations not determined) for 4 weeks. No surgery procedure was performed in this study. No significant differences in body weight at 4 weeks was observed in comparison with no treated rats. In the another toxicological study of tacrolimus in the Lewis rats with no surgery [42] led the tacrolimus therapy (4.0 mg/kg/day, peroral application, blood concentrations not determined) to initial loss of body weight in the 30 day follow-up period.

In the work of Azuma et al. resulted doses of 1.0 mg/kg/day (intramuscular application, blood concentrations not determined) in rats after aortal transplantation in adverse effects such as diarhea and weight loss. In the followed aortal transplantation experiments was the daily dosis of tacrolimus fixed at 0,2 mg/kg/day. However, no blood levels of tacrolimus was measured in this study as well [18].

In our first experimental work with tacrolimus immunosuppression after cold-stored abdominal aortic transplantation in rats was the daily dosis of tacrolimus given intramusculary fixed on 0.2 mg/kg/day. The plasma concentration of tacrolimus on day 30 after transplantation was 5.0 ± 0.7 ng/mL. This protocol showed good immunosuppressive effect on acute cell- and antibody-mediated rejection of cold-stored aortal grafts with no clinically manifested adverse effects [13,14].

Our clinical immunosuppressive protocol in patients with vascular prosthesis infection treated by transplantation of cold-stored arterial allografts consists of orally administered tacrolimus given from day 7 after transplantation. The usual starting dose of tacrolimus is 6 mg/day. The usual maintenance daily dose is 2 mg/day, respectively. The tacrolimus blood levels are determined periodically with blood concentration range between 4 and 7 ng/mL. No significant adverse effects of tacrolimus immunosuppression were observed in those patients [4].

In this study we confirmed possibility to delaying of low dose tacrolimus therapy after cryopreserved rat abdominal aortic transplantation for 7 days without negative effect on their acute cell- and antibody-mediated rejection. In the rat heart allograft transplantation model rescued tacrolimus (1.28 mg/kg, intramuscular application, blood concentrations not determined) given only on day four, five and six after transplantation all grafts and prolonged significantly survival of heart allografts [43]. In addition, in the rat cold-stored carotid transplantation model suppressed the low-dose of tacrolimus (0.2 mg/kg/day, intramuscular application, blood concentrations not determined) immunologic reaction even with administration starting on day 3 after transplantation [18]. However cessation of the use of tacrolimus led to severe rejection of transplanted carotids with dense cell infiltration in adventitia and medial degeneration within 14 days.

The possibility of delaying immunosuppression was studied in cold-stored thoracic aorta to abdominal aorta transplantation model in mice as well [30]. The monotherapy with everolimus (mammalian target of rampamycin inhibitor) reduced in daily doses of 0.05 mg/kg (intraperitoneal application, blood concentration of 10 ± 1.0 ng/mL) the formation of transplant arteriosclerosis on day 30 when therapy was only started on postoperative days 7 or 14. In addition, delayed combined treatment with everolimus (0.05 mg/kg/day, intraperitoneal application, blood concentration of 10 ±1.0 ng/mL) and clopidogrel (1 mg/kg/day, intraperitoneal application) administrated from day 7 or 14, reduced further the formation of transplant arteriosclerosis on day 30 after transplantation.

The antiplatelet therapy was in recipients of cryopreserved aortal allografts in our experiment not administered. However, in our clinical practice is the antiplatelet therapy in patients after cryopreserved arterial transplantation administered routinely.

In the end may this fact theoretically contribute to an increase in the immunosuppressive effect of tacrolimus in this specific patient population.

In the recently published work of Konrad H. et al was clearly demonstrated that cryopreserved arterial allografts used in clinical practice are highly immunogenic in terms of an HLA-directed immune response. This allogeneic immune response does not lead to an acute graft loss but to a chronic vascular degeneration process with clinically apparent thromboses with subsequent medical interventions up to amputations [9]. The low dose immunosuppression with sirolimus (mammalian target of rapamycine inhibitor) did not show any affective influence on antibody-mediated rejection of these cryopreserved arterial allografts. However, closely specification of this immunosuppressive protocol in towards to dosage, timing and blood concentration was not mentioned in this publication.

Our present study shows induction of donor specific anti MHC I and anti MHC II production in non-immunosuppressed recipients of cryopreserved aortal allografts during the 30 day follow-up period. Both immunosuppressive protocols with low-dose tacrolimus were sufficient to suppress this antibody production. The massive donor specific alloantibody response in mice recipients of cold-stored thoracic aortic allografts during the 30 day follow-up period was observed in the work of Heim et al. as well [30], The delayed therapy with everolimus alone (0.05 mg/kg/day, intraperitoneal application, blood concentration of 10 ±1.0 ng/mL) or in combinations with clopidogrel (1 mg/kg/day, intraperitoneal application) reduced the amount of donor-specific antibodies even if therapy was started on day 7 or on day 14. In our previous work we confirmed the suppressive effect of tacrolimus (0.2 mg/kg/day, intramuscular application from day 1 after transplantation, blood concentration of 5.57 ± 0.96 ng/mL) on donor specific antibody production in rats recipients of venous allografts as well [44].

In conclusion we have shown that low dose tacrolimus therapy was effective to suppress cell- and donor-specific antibody-mediated rejection of cryopreserved abdominal aortic allografts. We believe that clinical use of presented tacrolimus-based immunosuppressive protocol increase the success of the clinical program of cryopreserved alloarterial transplantation in the treatment of vascular prosthesis and stentgrafts infection in the Czech Republic.

## Supporting information

S1 File In this file there are all basic data regarding number of animals weight on the day 30, cold ischaemic time of transplanted grafts, cryoconservation time, thickness of tunica media on the day 30, number of CD 4 positive cells, as well as number of CD 8 positive cells, tacrolimus concentration and comparison between groups are available [S1 Table]. (XLS)
